# Editorial: Neural markers of sensory processing in development

**DOI:** 10.3389/fnint.2023.1256437

**Published:** 2023-07-21

**Authors:** Lauren E. Ethridge, Benjamin D. Auerbach, Anis Contractor, Iryna M. Ethell, Elizabeth A. McCullagh, Ernest V. Pedapati

**Affiliations:** ^1^Department of Psychology, University of Oklahoma, Norman, OK, United States; ^2^Department of Pediatrics, University of Oklahoma Health Sciences Center, Oklahoma City, OK, United States; ^3^Department of Molecular and Integrative Physiology, University of Illinois at Urbana-Champaign, Champaign, IL, United States; ^4^Department of Neuroscience, Psychiatry and Behavioral Sciences, and Neurobiology, Northwestern University, Evanston, IL, United States; ^5^Department of Biomedical Sciences, University of California, Riverside, Riverside, CA, United States; ^6^Department of Integrative Biology, Oklahoma State University, Stillwater, OK, United States; ^7^Department of Psychiatry and Behavioral Neuroscience, College of Medicine, University of Cincinnati, Cincinnati, OH, United States; ^8^Division of Child and Adolescent Psychiatry, and Child Neurology, Cincinnati Children's Hospital Medical Center, Cincinnati, OH, United States

**Keywords:** neurodevelopment, translational methods, EEG, neurodevelopmental disorders, sensory systems

## Introduction

Sensory processing dynamics have emerged as important contributors to typical cognitive and social development (Larkin et al., 2017; Cruz et al., 2022). In addition, sensory processing abnormalities are common in neurodevelopmental disorders, particularly in autism spectrum disorders (ASDs; Robertson and Baron-Cohen, 2017) and related genetic disorders like Fragile X Syndrome (FXS; Rais et al., 2018). This Research Topic explores the determinants of early sensory system maturation, functional consequences of changes to sensory development, and methodologies to investigate under-studied modalities of sensation to further the understanding of the role of sensory systems in a range of functional outcomes.

## Electroencephalography (EEG) in sensory systems research

EEG is a frequently employed method in the translational neuroscience of sensory systems. EEG provides a direct measure of neural oscillatory patterns on a millisecond time-scale consistent with the speed at which sensory systems operate to detect and process information. EEG is safe and well-tolerated, and data can be readily collected from infant and clinical populations while awake and performing a task. Local field potentials, such as measured in aggregate with EEG, are easily bridged to animal models for circuit-level investigation of neural findings conserved across species. An et al. found that children demonstrated minimal habituation to repeated tones; however, improved language ability in children with FXS was associated with increased habituation in the frontal cortex. The habituation task, which shows conservation across species in FXS (Lovelace et al., 2016) is thought to represent a proxy of auditory hypersensitivity (Ethridge et al., 2016), and the findings suggest that this aspect of auditory system maturation is more critical in atypical than typical development for language acquisition.

## Sensory processing changes in NDDs

Subtle sensory processing changes may mediate atypical developmental trajectories even in neurodevelopmental disorders where sensory symptoms are not at the forefront, or may be indicative of neural changes related to early developmental events. Witteveen et al. examined the *1/f* aperiodic slope of EEG spectrograms in healthy infants as a proxy for maturation and found advanced maturation was consistent with birth, rather than gestational age, triggering changes in visual cortex activity. These effects were recapitulated in local field potential recordings of preterm mice, driven by enhanced inhibition via changes to distribution of parvalbumin expression levels in PV-expressing interneurons. This conservation of visual cortical activity with preterm birth suggests that maturation of the power spectrum in primary visual cortex may be an important indicator of sensory system development relevant to future assessment of neurocognitive outcomes. It is, however, unclear at this point whether these changes to visual system maturation are driven by environmental changes (birth) alone, or by other factors contributing to increased chance of preterm birth.

Much work has been done in this area describing typical development in humans and animal models for visual and auditory modalities, however, gaps remain in understanding how these measures correspond across species over various developmental periods. Significant gaps also persist in describing the development of these measures in sensory modalities beyond vision and audition. In particular, the ability to deliver visual and auditory stimuli with high precision may partially account for the preponderance of neuroimaging and EEG developmental studies in the visual and auditory domains, rather than enhanced importance of these domains to developmental outcomes (Espenhahn et al., 2021; Ahlfors et al., 2022; Arpaia et al., 2022). Bhattacharjee et al. describe the development of a novel tactile stimulus delivery system to enable measurement of active somatosensation with the precision necessary to enable use in EEG, magnetoencephalography (MEG), and other neurophysiology studies. Tactile hypersensitivity is commonly reported in ASDs, but the neural correlates and development of the tactile system is severely understudied in ASD (Marco et al., 2011). Technological enhancements like the ones reported here will enable researchers to provide a more balanced investigation of sensory systems and their neural correlates across both typical and atypical development.

Finally, it is important to note that studying the neural correlates of sensory systems is critically hampered by the lack of psychometrically sound measures for characterizing sensory behaviors in both humans and animal models. This is a strong limitation to what degree we can map clinical alterations in sensation to brain activity. Mulligan et al., describe overlapping phenotypes in children with idiopathic sensory processing disorder, with the majority of children showing a combination of multiple sub-types, suggesting that current methodology for classifying sensory behaviors in humans requires further study to develop categories useful for assessing developmental trajectories and outcomes and for mapping to neural measures.

In summary, understanding sensory system development is of critical importance for predicting functional outcomes across a variety of linked constructs in both neurodevelopmental disorders and typical development. Translational work using methodologies such as EEG can provide significant insight into neural correlates of sensory processing, and is feasible in both developmental human populations and animal models, allowing further study into circuit and molecular mechanisms for sensory system modulation. However, study in this area is limited by technological advancements in some sensory modalities more than others, and a lack of strong assessment methodology for quantifying sensory behaviors. Advancement in these areas is critically needed to incorporate already translationally sophisticated neuroimaging and neurophysiological methods into a comprehensive view of sensory system development.

## Author contributions

All authors listed have made a substantial, direct, and intellectual contribution to the work and approved it for publication.

## References

[B1] Ahlfors S. P. Graham S. Alho J. Joseph R. M. McGuiggan N. M. Nayal Z.. (2022). Magnetoencephalography and electroencephalography can both detect differences in cortical responses to vibrotactile stimuli in individuals on the autism spectrum. Front. Psychiatry 13, 902332. 10.3389/fpsyt.2022.902332 35990048 PMC9388788

[B2] Arpaia P. Cataldo A. Criscuolo S. De Benedetto E. Masciullo A. Schiavoni R.. (2022). Assessment and scientific progresses in the analysis of olfactory evoked potentials. Bioengineering 9, 252. 10.3390/bioengineering9060252 35735495 PMC9219708

[B3] Cruz S. Lifter K. Barros C. Vieira R. Sampaio A. (2022). Neural and psychophysiological correlates of social communication development: evidence from sensory processing, motor, cognitive, language and emotional behavioral milestones across infancy. Appl. Neuropsychol. Child 11, 158–177. 10.1080/21622965.2020.1768392 32449376

[B4] Espenhahn S. Godfrey K. J. Kaur S. Ross M. Nath N. Dmitrieva O.. (2021). Tactile cortical responses and association with tactile reactivity in young children on the autism spectrum. Mol. Autism. 12, 26. 10.1186/s13229-021-00435-9 33794998 PMC8017878

[B5] Ethridge L. E. White S. P. Mosconi M. W. Wang J. Byerly M. J. Sweeney J. A.. (2016). Reduced habituation of auditory evoked potentials indicate cortical hyper-excitability in Fragile X Syndrome. Transl. Psychiatry 6, e787. 10.1038/tp.2016.48 27093069 PMC4872406

[B6] Larkin F. Meins E. Centifanti L. C. M. Fernyhough C. Leekam S. R. (2017). How does restricted and repetitive behavior relate to language and cognition in typical development? Dev. Psychopathol. 29, 863–874. 10.1017/S0954579416000535 27417193

[B7] Lovelace J. W. Wen T. H. Reinhard S. Hsu M. S. Sidhu H. Ethell I. M.. (2016). Matrix metalloproteinase-9 deletion rescues auditory evoked potential habituation deficit in a mouse model of Fragile X Syndrome. Neurobiol. Dis. 89, 126–135. 10.1016/j.nbd.2016.02.002 26850918 PMC4785038

[B8] Marco E. J. Hinkley L. B. Hill S. S. Nagarajan S. S. (2011). Sensory processing in autism: a review of neurophysiologic findings. Pediatr. Res. 69, 48R−54R. 10.1203/PDR.0b013e3182130c54 21289533 PMC3086654

[B9] Rais M. Binder D. K. Razak K. A. Ethell I. M. (2018). Sensory processing phenotypes in fragile X syndrome. ASN Neuro. 10, 1759091418801092. 10.1177/1759091418801092 30231625 PMC6149018

[B10] Robertson C. E. Baron-Cohen S. (2017). Sensory perception in autism. Nat. Rev. Neurosci. 18, 671–684. 10.1038/nrn.2017.112 28951611

